# Albumin-Corrected Serum Calcium as a Biochemical Correlate of Left Ventricular Dysfunction Among Patients With End-Stage Renal Disease on Maintenance Hemodialysis: A Cross-Sectional Study

**DOI:** 10.7759/cureus.114664

**Published:** 2026-08-17

**Authors:** Biswajit Pattnaik, Sanjay Kumar Jangid, Kalankikar Chand, Bhabani S Samantaray, Ashutosh Das

**Affiliations:** 1 Department of General Medicine, Hi-Tech Medical College and Hospital, Bhubaneswar, IND; 2 Department of Pharmacology, Roland Institute of Pharmaceutical Sciences, Berhampur, IND

**Keywords:** albumin-corrected calcium, ejection fraction, end-stage renal disease, hemodialysis, left ventricular dysfunction, serum albumin

## Abstract

Background: Cardiovascular disease is the leading cause of mortality in end-stage renal disease (ESRD), with left ventricular dysfunction (LVD) as its most consequential manifestation. Despite the well-established role of disturbed mineral metabolism in uremic cardiomyopathy, the calcium-phosphate (Ca×PO₄) product is widely used as a composite risk marker in ESRD management. This study aimed to determine the prevalence of LVD in ESRD patients on maintenance hemodialysis and to evaluate albumin-corrected calcium, Ca×PO₄ product, serum albumin, hemoglobin, and uric acid as cross-sectional correlates of left ventricular ejection fraction (LVEF). Sixty consecutive ESRD patients on stable thrice-weekly hemodialysis underwent predialysis transthoracic echocardiography, and LVD was defined as LVEF <50%. Total serum calcium was corrected for albumin using the Payne formula.

Results: LVD was present in 58.3% of patients (35/60); 35.0% met criteria for heart failure with reduced ejection fraction (LVEF <40%) and 23.3% for heart failure with mildly reduced ejection fraction (40%-49%). Among all biochemical parameters evaluated, albumin-corrected calcium was the sole independent associate of LVD on multivariate analysis (adjusted OR, 5.83; 95% CI, 1.84-18.42; p = 0.003), although causal inference is precluded by the cross-sectional design. Serum albumin demonstrated the strongest univariate biochemical correlation with LVEF (Spearman r = 0.701, p <0.001), exceeding corrected calcium (r = -0.488, p <0.001). The Ca×PO₄ product, in both uncorrected (r = -0.121, p = 0.358) and corrected form (r = -0.129, p = 0.329), did not predict LVD. Hemoglobin and uric acid similarly showed no significant association with LVEF. E/e' ratio was the strongest echocardiographic correlate of LVEF (r = -0.566, p <0.001) and was significantly elevated in the LVD group (median 13.9 vs. 10.8; p = 0.001).

Conclusions: LVD is highly prevalent in ESRD patients on maintenance hemodialysis. Ca×PO₄ product does not show significant cross-sectional association with left ventricular systolic dysfunction in either corrected or uncorrected form. Albumin-corrected calcium was a key independent biochemical correlate of LVD in this cohort, while serum albumin emerged as an additional strong cardiac risk correlate warranting prospective evaluation. However, longitudinal studies are needed to determine whether these associations are causal and to inform clinical practice.

## Introduction

Chronic kidney disease (CKD) affects approximately 10%-15% of adults worldwide, with a substantial number progressing to end-stage renal disease (ESRD) requiring renal replacement therapy [1]. Despite the life-sustaining role of maintenance hemodialysis, dialysis-dependent patients continue to have disproportionately high cardiovascular morbidity and mortality, with cardiovascular disease accounting for approximately 50% of all-cause mortality in this population, a risk 10- to 30-fold higher than in the general population [2,3].

Left ventricular (LV) structural and functional abnormalities are central to this cardiovascular burden. Uremia drives a spectrum of LV changes that include hypertrophy, dilation, systolic dysfunction, and diastolic dysfunction, which are further accelerated by hemodynamic overload, uremic toxins, and dysregulated mineral metabolism [4]. Reduction in left ventricular ejection fraction (LVEF) is a powerful independent predictor of all-cause and cardiovascular mortality in hemodialysis patients [5].

Among the metabolic derangements of ESRD, disturbances of calcium homeostasis have attracted particular pathophysiological interest. In the hemodialysis setting, serum calcium reflects not only dietary intake but also a complex interplay of calcium-based phosphate binder use, active vitamin D analog therapy, and secondary hyperparathyroidism [6]. Calcium excess promotes vascular and valvular calcification, increases arterial stiffness and systolic afterload, and has been directly associated with myocardial dysfunction and intradialytic hemodynamic instability [7,8]. The composite calcium-phosphate (Ca×PO₄) product has historically served as a surrogate marker for mineral dysregulation in ESRD, with Kidney Disease: Improving Global Outcomes (KDIGO) guidelines recommending its maintenance below 55 mg²/dL [9]. However, this composite metric may obscure the differential contributions of its individual components, particularly when calcium and phosphate move discordantly in treated hemodialysis patients.

Other hematological and biochemical parameters implicated in LV remodeling include anemia, which drives eccentric hypertrophy through volume overload, and serum uric acid, associated with LV hypertrophy and diastolic dysfunction in nonuremic populations through oxidative stress and endothelial dysfunction, although its independent role in the uniformly hyperuricemic ESRD population remains uncertain [10,11].

Data systematically evaluating serum calcium as an independent correlate of LV systolic dysfunction, distinct from the composite Ca×PO₄ product, are limited, particularly from Indian tertiary care centers. This study aimed to determine the prevalence and echocardiographic profile of LV dysfunction in ESRD patients on maintenance hemodialysis and to identify independent biochemical correlates of LVEF, with specific attention to the differential contributions of serum calcium, Ca×PO₄ product, and hemoglobin.

## Materials and methods

Study design and setting

A cross-sectional observational study was conducted in the Department of General Medicine at a tertiary care center in Odisha, India, between October 2025 and July 2026. Ethical approval was obtained from the Institutional Ethics Committee, and written informed consent was obtained from all participants prior to enrolment. The study was conducted in accordance with the ethical principles of the Declaration of Helsinki.

Study participants

**Figure 1:**
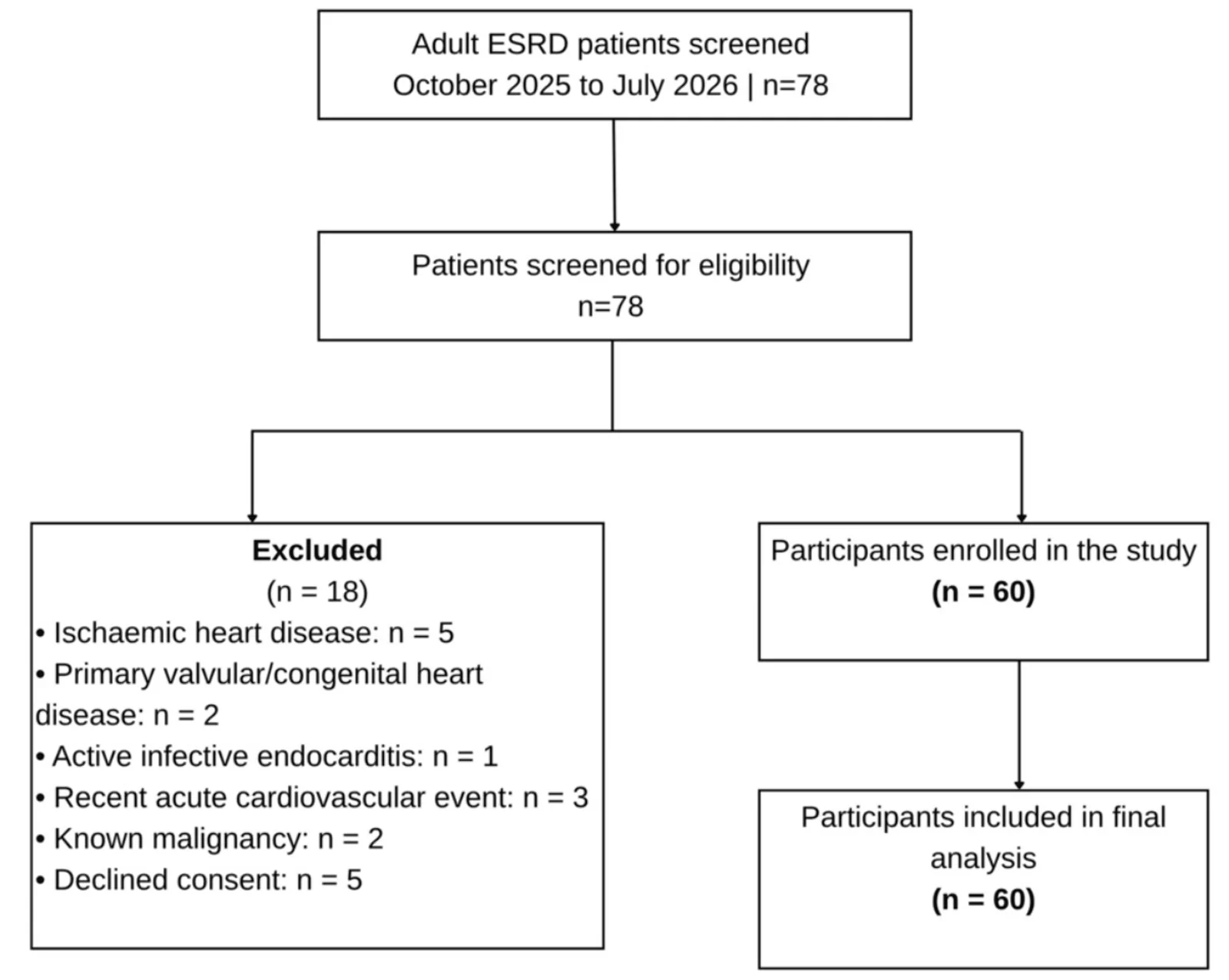
Flow diagram showing participant recruitment, eligibility assessment, exclusions, enrolment, and inclusion in the final analysis ESRD: end-stage renal disease

Patients were recruited consecutively between October 2025 and July 2026. Of 78 adult ESRD patients on maintenance hemodialysis screened during this period, 18 were excluded (five with ischemic heart disease, two with primary valvular/congenital heart disease, one with active infective endocarditis, three with a recent acute cardiovascular event, two with known malignancy, and five who declined consent), yielding a final sample of 60 participants (Figure 1).

Clinical and biochemical assessment

Demographic data including age, sex, and duration of hemodialysis were recorded. Comorbid conditions including type 2 diabetes mellitus (T2DM) and hypertension were documented from case records. Blood samples were collected before the mid-week hemodialysis session. Serum biochemistry, including urea, creatinine, uric acid, sodium, potassium, calcium, and phosphate, was measured using standard automated analyzers in the institutional laboratory. Glomerular filtration rate (GFR) was estimated using the CKD Epidemiology Collaboration creatinine equation [12].

Since serum albumin is frequently depressed in ESRD due to chronic inflammation, protein loss, and nutritional deficiency, total serum calcium was corrected for serum albumin concentration using the following Payne formula [13]:

$\text{Corrected calcium (mg/dL)} {=} \text{Measured total calcium (mg/dL)} {+} 0.8 {\times} [4.0 {-} \text{Serum albumin (g/dL)}]$.

This correction was applied uniformly across all participants prior to statistical analysis, as uncorrected total calcium systematically underestimates ionized calcium bioavailability in hypoalbuminemic patients and may attenuate the true association between calcium and cardiac outcomes. It is acknowledged that albumin-based correction formulas have recognized accuracy limitations in ESRD, where disturbances in protein binding, acid-base balance, and mineral metabolism deviate from the assumptions of the linear Payne formula; ionized calcium measurement, which would directly reflect physiologically active calcium, was not available at this center. All calcium values reported in this study, including group comparisons, Spearman correlations, and logistic regression, refer to albumin-corrected calcium unless otherwise stated.

Echocardiographic assessment

All patients underwent transthoracic echocardiography performed by a trained cardiologist within 30 minutes before the midweek hemodialysis session to minimize the confounding effects of acute fluid shifts. The following parameters were recorded: LVEF by the modified Simpson biplane method, left atrial (LA) diameter, LV internal diameter in diastole (LVIDd) and LV internal diameter in systole (LVIDs), and right ventricular (RV) dimension. LV morphology, valvular abnormalities, pericardial effusion, and the presence of pulmonary arterial hypertension were also noted. Ejection fraction (EF) was classified according to American College of Cardiology (ACC)/American Heart Association (AHA) 2022 Heart Failure Guidelines: heart failure with reduced ejection fraction (HFrEF) (<40%), heart failure with mildly reduced ejection fraction (HFmrEF) (40%-49%), and heart failure with preserved ejection fraction (HFpEF) (≥50%) [14]. LVD was defined as EF <50% for the primary outcome of this study.

Ethical considerations

Ethical approval was obtained from the Institutional Ethics Committee, Hi-Tech Medical College and Hospital, Bhubaneswar (Approval No. HMCH/IEC/2025/90, dated October 25, 2025) for the study.

Data were analyzed using Statistical Package for the Social Sciences, version 28 (IBM Corp., Armonk, NY). Continuous variables were tested for normality using the Shapiro-Wilk test; as most variables followed a nonnormal distribution, results are expressed as median (interquartile range (IQR)) unless otherwise specified. Categorical variables are presented as frequencies and percentages. Between-group comparisons (LVD vs. no LVD) were performed using the Mann-Whitney U test for continuous variables and the chi-squared test (or Fisher's exact test for cell frequencies <5) for categorical variables. Spearman rank correlation was used to assess the relationship between biochemical and structural echocardiographic parameters and EF. Binary logistic regression was performed with LVD as the dependent variable; variables significant in univariate analysis (p < 0.05) were entered into the multivariate model. A two-tailed p value of <0.05 was considered statistically significant throughout.

## Results

Baseline characteristics

Sixty patients were included, of whom 40 (66.7%) were male and 20 (33.3%) were female patients. LVD (EF < 50%) was present in 35 patients (58.3%). Median age was 53 years (IQR, 46-64); patients with LVD were significantly older than those without (61 vs. 49 years, p = 0.005). T2DM was present in eight patients (13.3%), with no significant association with LVD (p = 0.449). Hypertension was significantly more common in the non-LVD group (32.0% vs. 5.7%, p = 0.012). Sex distribution (p = 0.517) and hemoglobin levels (p = 0.462) were comparable between the groups. Baseline characteristics are summarized in Table 1.

**Table 1:** Baseline characteristics of study participants stratified by LVD status ^†^Mann-Whitney U test ^#^Fisher's exact test LVD: left ventricular dysfunction; EF: ejection fraction; IQR: interquartile range; T2DM: type 2 diabetes mellitus; GFR: glomerular filtration rate

Variable	Total (n = 60)	LVD (EF < 50%; n = 35)	No LVD (EF ≥ 50%; n = 25)	p value
Age (years), median (IQR)	53 (46-64)	61 (49-67)	49 (42-54)	0.005^†^
Male sex, n (%)	40 (66.7%)	25 (71.4%)	15 (60.0%)	0.517^#^
Female sex, n (%)	20 (33.3%)	10 (28.6%)	10 (40.0%)	0.517^#^
T2DM, n (%)	8 (13.3%)	6 (17.1%)	2 (8.0%)	0.449^#^
Hypertension, n (%)	10 (16.7%)	2 (5.7%)	8 (32.0%)	0.012^#^
Hemoglobin (g/dL), median (IQR)	9.1 (8.4-9.5)	9.1 (8.1-9.5)	9.1 (8.4-9.7)	0.462^†^
GFR (mL/minute)	8.5 (6.0-12.2)	9.2 (6.9-11.8)	7.0 (5.0-13.3)	0.280^†^

Prevalence and classification of LVD

**Figure 2:**
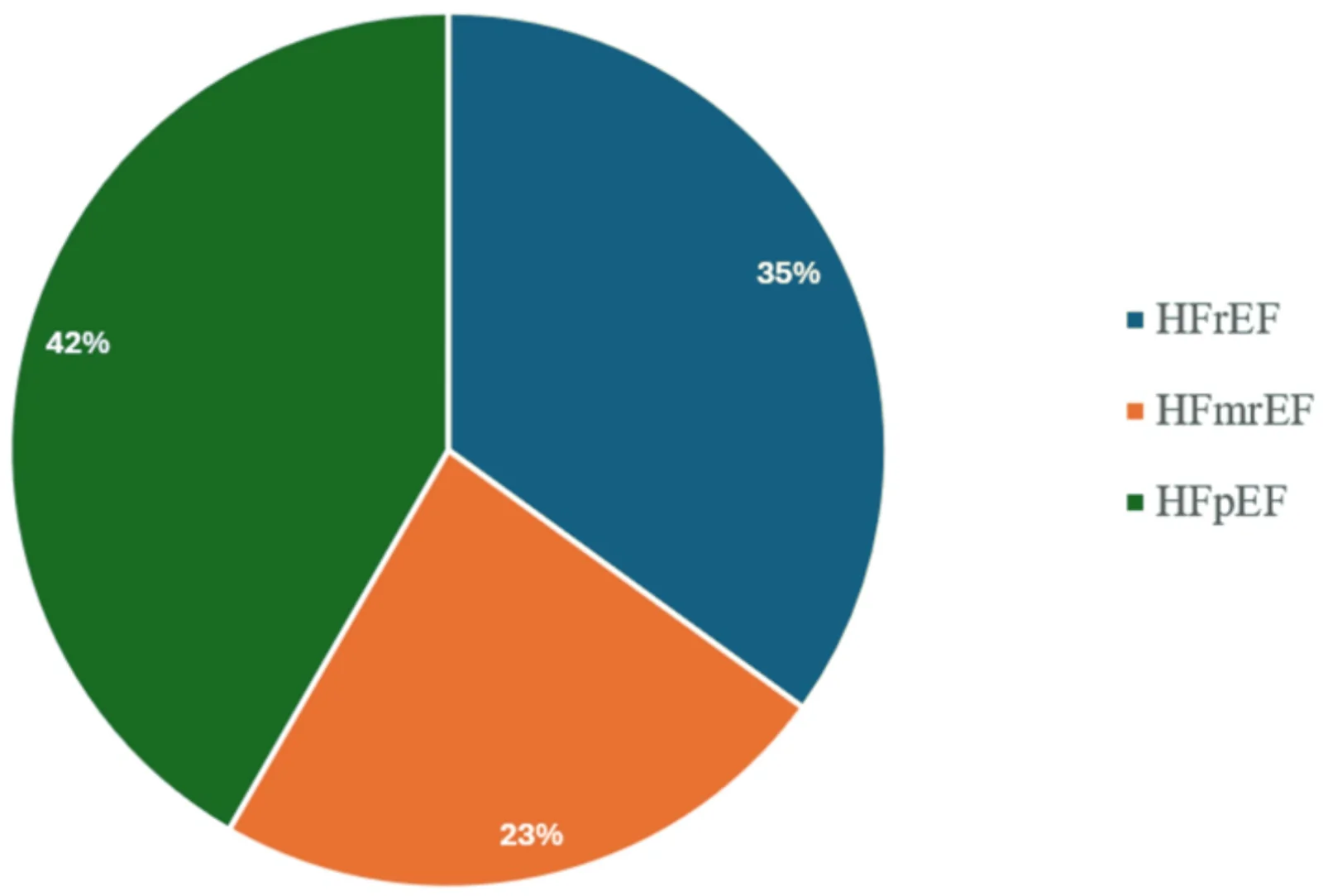
Pie chart showing distribution of patients by EF category HFmrEF: heart failure with mildly reduced ejection fraction; HFpEF: heart failure with preserved ejection fraction; HFrEF: heart failure with reduced ejection fraction; EF: ejection fraction

Left ventricular dysfunction (LVD) was defined as an EF <50%. Applying the 2022 ACC/AHA EF-based thresholds to categorize systolic function, 21 patients (35.0%) had EF <40% (consistent with HFrEF), and 14 patients (23.3%) had EF 40%-49% (consistent with HFmrEF); together, these 35 patients (58.3%) constituted the LVD group. The remaining 25 patients (41.7%) had preserved EF (≥50%) and did not meet the study's definition of LVD. As this was a cross-sectional echocardiographic study without formal clinical assessment for heart failure signs or symptoms, the EF <40% and 40%-49% categories are reported as echocardiographic correlates of HFrEF/HFmrEF rather than confirmed heart failure diagnoses, and the preserved-EF group is described as such rather than as HFpEF. The distribution of patients by EF category is presented in Table 2 and Figure 2.

**Table 2:** Distribution of patients by EF category EF: ejection fraction

Category	EF range	n (%)
Heart failure with reduced EF	<40%	21 (35.0%)
Heart failure with mildly reduced EF	40-49%	14 (23.3%)
Heart failure with preserved EF	≥50%	25 (41.7%)

Biochemical and hematological parameters and their correlation with EF

**Figure 3:**
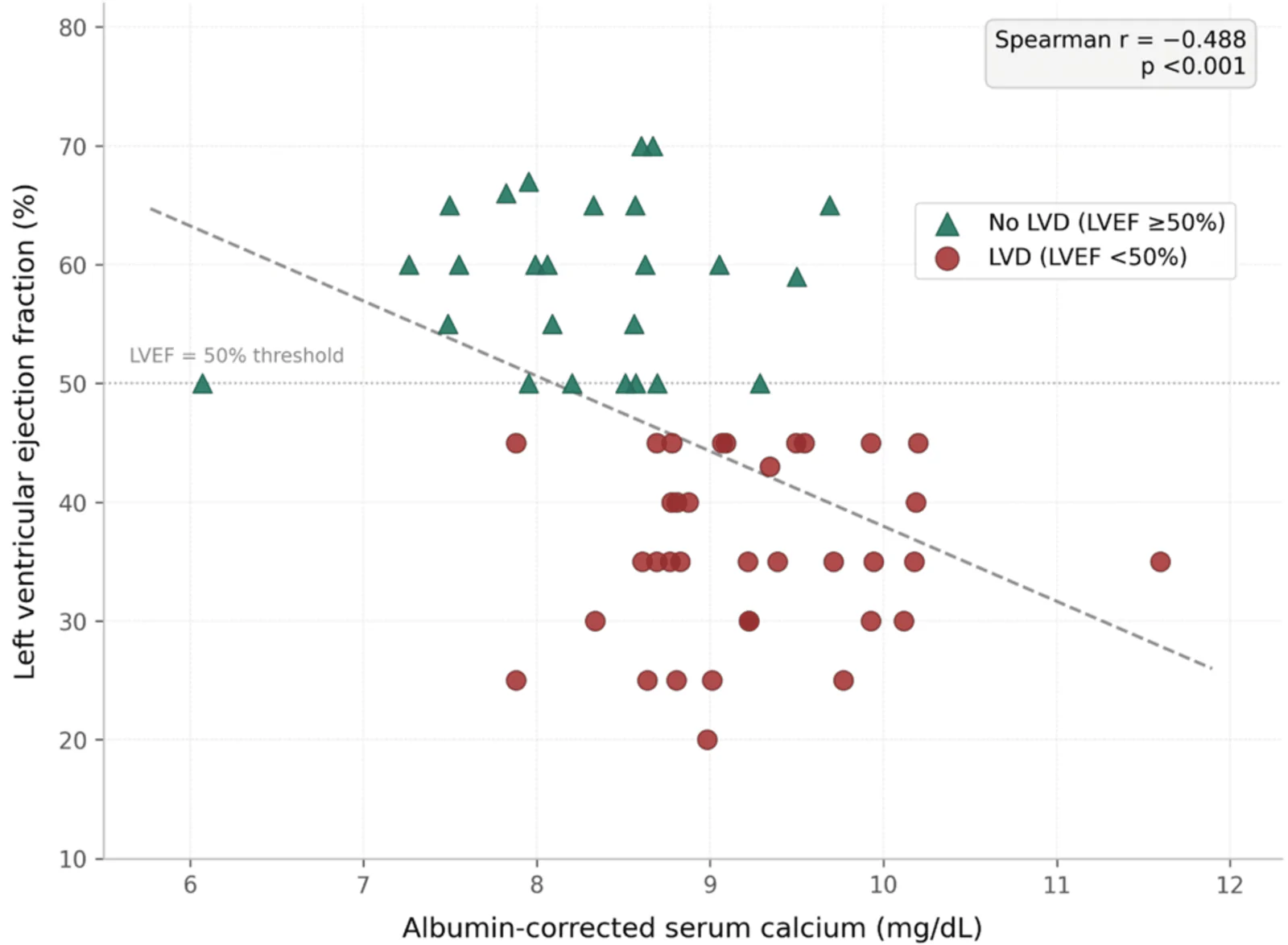
Scatter plot of albumin-corrected serum calcium vs. LVEF in study population Each point represents one patient; red circles indicate patients with left ventricular dysfunction (LVEF <50%) and green triangles indicate patients without left ventricular dysfunction (LVEF ≥50%). The dashed line represents the linear regression fit. The dotted horizontal line denotes the LVEF 50% threshold used to define left ventricular dysfunction. Spearman r = -0.488, p <0.001 LVEF: left ventricular ejection fraction; LVD: left ventricular dysfunction

Table 3 summarizes the biochemical and hematological parameters of the study population and their relationship with LVEF. Serum albumin, total calcium, and albumin-corrected calcium showed significant associations with EF (Table 3). Albumin correlated positively with EF (r = 0.701, p < 0.001), while total calcium (r = -0.348, p < 0.001) and albumin-corrected calcium (r = -0.488, p < 0.001) correlated inversely, indicating lower EF with higher calcium levels (Figure 3). Patients with LVD had lower albumin and higher calcium than those without. Urea, creatinine, GFR, sodium, potassium, uric acid, phosphate, and Ca×PO₄ product showed no significant correlation with EF and did not differ between the groups. Serum albumin and calcium-related parameters were thus the only biochemical markers significantly associated with LV systolic function in this cohort.

**Table 3:** Biochemical and hematological parameters: correlation with EF and comparison between the LVD and non-LVD groups ^†^Mann-Whitney U test LVD: left ventricular dysfunction; EF: ejection fraction

Parameter	Total (n = 60)	LVD (n = 35)	No LVD (n = 25)	Spearman r	p (group)^†^
Urea (mg/dL)	117.7 (97.3-182.9)	118.4 (107.8-179.4)	109.7 (91.0-199.3)	-0.179	0.315
Creatinine (mg/dL)	6.4 (4.3-9.3)	5.5 (4.7-7.6)	7.9 (3.8-10.7)	0.109	0.301
Uric acid (mg/dL)	7.6 (6.1-8.8)	7.5 (6.0-8.7)	8.2 (6.5-8.7)	0.040	0.538
Sodium (mEq/L)	134.5 (130.0-137.9)	134.1 (130.0-137.8)	135.4 (131.5-137.9)	0.004	0.527
Potassium (mEq/L)	4.8 (4.3-5.4)	4.9 (4.3-5.5)	4.7 (4.3-5.3)	0.028	0.697
Serum albumin (g/dL)	2.8 (2.6-3.0)	2.6 (2.6-2.8)	3.0 (2.9-3.1)	0.701	<0.001
Serum calcium, total (mg/dL)	7.8 (7.5-8.4)	8.1 (7.7-8.6)	7.6 (7.1-7.8)	-0.348	<0.001
Albumin-corrected calcium (mg/dL)	8.8 (8.3-9.4)	9.1 (8.8-9.7)	8.3 (8.0-8.6)	-0.488	<0.001
Serum phosphate (mg/dL)	6.4 (5.0-7.6)	6.2 (5.1-7.3)	6.5 (4.8-8.2)	0.038	0.668
Ca×PO₄ product, uncorrected (mg²/dL²)	48.3 (40.7-59.0)	49.9 (42.2-59.2)	45.8 (39.7-56.2)	-0.121	0.315
Ca×PO₄ product, corrected (mg²/dL²)	54.3 (45.4-65.3)	56.8 (47.2-66.7)	50.5 (43.4-63.1)	-0.129	0.241

Echocardiographic parameters

E/e' ratio showed the strongest echocardiographic correlation with EF (r = -0.566, p < 0.001) and was significantly elevated in the LVD group (median 13.9 vs. 10.8, p = 0.001), confirming concurrent diastolic dysfunction. Among structural parameters, LVIDs showed the strongest correlation with EF (r = -0.479, p < 0.001), followed by LVIDd (r = -0.425, p = 0.001), RV dimension (r = -0.282, p = 0.029), and LA diameter (r = -0.270, p = 0.037). LVIDd and LVIDs were significantly larger in the LVD group, confirming LV dilation as the dominant structural substrate of systolic dysfunction. Data are presented in Table 4.

**Table 4:** Echocardiographic parameters stratified by LVD status and correlation with EF Mann-Whitney U test was performed LVD: left ventricular dysfunction; EF: ejection fraction; LA: left atrial; LVIDd: left ventricular internal diameter in diastole; LVIDs: left ventricular internal diameter in systole; RV: right ventricular

Parameter	Total (n = 60)	LVD (n = 35)	No LVD (n = 25)	Spearman r	p value
EF (%)	45 (35-56)	35 (28-43)	61 (57-66)	-	-
E/e' ratio	12.2 (10.2-15.0)	13.9 (11.7-16.1)	10.8 (9.8-12.1)	-0.566	<0.001
LA diameter (mm)	39 (35-42)	40 (36-44)	38 (34-42)	-0.270	0.037
LVIDd (mm)	48.5 (43.9-53.9)	50.8 (46.4-56.0)	46.0 (41.0-51.0)	-0.425	0.001
LVIDs (mm)	34.5 (30.4-42.6)	41.5 (36.5-47.0)	31.7 (27.0-35.5)	-0.479	<0.001
RV dimension (mm)	18 (17-28)	18 (17-28)	17 (17-17)	-0.282	0.029

Multivariate logistic regression: independent predictors of LV dysfunction

Age, albumin-corrected calcium, and hypertension were entered into multivariate binary logistic regression based on univariate significance. Albumin-corrected calcium remained the only independent correlate of LVD (adjusted OR, 5.828; 95% CI, 1.844-18.419; p = 0.003). Hypertension showed a protective trend that did not reach significance (adjusted OR, 0.206; 95% CI, 0.035-1.220; p = 0.082), and age was not independently associated with LVD after adjustment (p = 0.429). Albumin-corrected calcium thus emerged as the strongest independent determinant of LV systolic dysfunction in this cohort. Results are presented in Table 5.

**Table 5:** Multivariate binary logistic regression: independent predictors of LVD (EF <50%) LVD: left ventricular dysfunction; EF: ejection fraction

Variable	β	Crude OR (95% CI)	Adjusted OR (95% CI)	p value
Age (years)	0.022	1.060 (1.013-1.110)	1.023 (0.968-1.081)	0.429
Albumin-corrected calcium (mg/dL)	1.763	7.075 (2.349-21.314)	5.828 (1.844-18.419)	0.003
Hypertension	-1.581	0.154 (0.031-0.753)	0.206 (0.035-1.220)	0.082

## Discussion

This cross-sectional study evaluated LV dysfunction and its biochemical correlates in 60 ESRD patients on maintenance hemodialysis. CKD affects a substantial proportion of the global population [1], and access to renal replacement therapy remains inequitable worldwide [2], underscoring the need for low-cost, accessible cardiac risk markers in resource-limited dialysis settings such as ours. LVD (EF <50%) was present in 58.3% of patients, with over a third meeting HFrEF criteria. Albumin-corrected calcium, not the Ca×PO₄ product, emerged as the key biochemical determinant of systolic dysfunction.

A large retrospective study of 1,019 hemodialysis patients reported comparable LVEF abnormality prevalence, with higher mortality and cardiovascular events in HFrEF/HFmrEF subgroups [3], consistent with regional data reporting LV systolic dysfunction in 31%-59% of South Asian dialysis cohorts [4]. This burden, comparable to trends in US dialysis populations [5], supports routine echocardiographic surveillance at hemodialysis initiation, not yet standard in Indian dialysis units. The older age of LVD patients aligns with established age- and vintage-related cardiovascular risk accrual [6].

The Ca×PO₄ product showed no correlation with LVEF, uncorrected (r = -0.121, p = 0.358) or corrected (r = -0.129, p = 0.329), despite being within KDIGO's target range [7]. Corrected calcium was elevated in the LVD group while phosphate was not, indicating the product's null result masked a calcium-driven signal, a limitation of composite metrics when components diverge, as occurs with concurrent calcium-based binder and vitamin D analog use [8].

Albumin-corrected calcium was the sole independent correlate of LVD (adjusted OR, 5.828; 95% CI, 1.844-18.419; p = 0.003). This is biologically coherent: iatrogenic-positive calcium balance in ESRD [8] drives vascular calcification, arterial stiffness, and afterload [9], with valvular calcification adding mechanical burden [10]; interventional dialysate-calcium studies directly demonstrate that higher dialysate calcium concentration worsens LV function on serial echocardiography [11], reinforcing a causal pathway consistent with our cross-sectional finding. The stronger correlation with corrected vs. total calcium (r = -0.488 vs. -0.348) reflects the Payne correction's role in unmasking bioavailable calcium in hypoalbuminemic patients [14]. Notably, both extremes of serum calcium carry cardiac risk in this population: hypercalcemia promotes vascular and valvular calcification and pressure overload as described above, while hypocalcemia is independently linked to increased QT dispersion, prolonged QT interval, and sudden cardiac death, underscoring calcium's narrow therapeutic window in dialysis patients and the debate over optimal dialysate calcium concentration [15]. This is consistent with data from a large ICU heart failure cohort (eICU Collaborative Research Database, n = 11,373) demonstrating a nonlinear, threshold-dependent relationship between serum calcium and cardiac arrest risk, with risk rising sharply above 9.5 mg/dL, a pattern that persisted using albumin-corrected calcium on sensitivity analysis [16].

Notably, the direction of our finding contrasts with reports in nonuremic populations: a large Chinese cohort of nearly 6,000 coronary artery disease patients found low, not high, serum calcium independently associated with LV systolic dysfunction and myocardial infarction [17]. This divergence likely reflects the distinct pathophysiology of ESRD, where hypercalcemia is largely iatrogenic and calcification-driven rather than reflecting acute ischemic calcium flux, and underscores that calcium-cardiac relationships established in nonrenal populations cannot be extrapolated to dialysis patients.

Serum albumin itself showed the strongest univariate correlation with EF (r = 0.701, p < 0.001). Sensitivity analysis confirmed albumin retained independent significance (p = 0.003) while total calcium did not (p = 0.114), suggesting hypoalbuminemia, a marker of inflammation and protein-energy wasting, contributes substantially to the corrected-calcium signal, likely bidirectionally [6].

Hemoglobin (median 9.1 g/dL) and uric acid showed no association with LVEF, plausibly reflecting ceiling effects from near-universal anemia and hyperuricemia rather than absence of the mechanistic roles established elsewhere [6,18]. Hypertension's paradoxical inverse univariate association with LVD, not retained on adjustment (p = 0.082), likely reflects survivor/ascertainment bias, also reported elsewhere [3]. E/e' ratio was the strongest echocardiographic correlate (r = -0.566, p < 0.001), consistent with known coupling of systolic and diastolic dysfunction via shared hemodynamic pathways [19].

In summary, LVD is highly prevalent in this cohort, and albumin-corrected calcium is its key independent biochemical correlate, with serum albumin an important contributing factor. Prospective multicenter studies incorporating parathyroid hormone (PTH), dialysis vintage, dialysate calcium, and cardiac biomarkers are needed to establish causality.

This single-center, cross-sectional study of 60 patients limits generalizability; Firth regression addressed small-sample separation, although CIs remain wide. Key confounders (PTH, fibroblast growth factor-23 (FGF-23), vitamin D, dialysis adequacy) and cardiac biomarkers were unmeasured, and corrected calcium was estimated rather than measured as ionized calcium.

## Conclusions

In our cross-sectional study of 60 patients, the Ca×PO₄ product showed no significant association with LV systolic dysfunction, contrary to our primary hypothesis. Albumin-corrected calcium was the only independent biochemical correlate of LVD on multivariate analysis. This suggests individual mineral metabolism markers may be more informative than the composite Ca×PO₄ product, although the cross-sectional design cannot establish causality or timing between corrected calcium and LVD. Serum albumin also showed a strong univariate association with LVEF, pointing to hypoalbuminemia as a clinically relevant cardiac risk marker worth prospective study. Overall, these hypothesis-generating findings suggest that monitoring corrected calcium and albumin separately, alongside routine echocardiography (LVEF and E/e'), may help identify cardiac risk in hemodialysis patients. Larger, longitudinal, multicenter studies incorporating ionized calcium, PTH, FGF-23, and dialysis vintage are needed to confirm causality and determine whether correcting these parameters improves cardiac outcomes.
